# 3D printed energy devices: generation, conversion, and storage

**DOI:** 10.1038/s41378-024-00708-2

**Published:** 2024-07-02

**Authors:** Jin-ho Son, Hongseok Kim, Yoonseob Choi, Howon Lee

**Affiliations:** Department of Mechanical Engineering, Institute of Advanced Machines and Design, Seoul National University, Seoul, Republic of Korea

**Keywords:** 3D printing, 3D-printed energy device, Energy generation device, Energy conversion device, Energy storage device, Electrical and electronic engineering, Materials science

## Abstract

The energy devices for generation, conversion, and storage of electricity are widely used across diverse aspects of human life and various industry. Three-dimensional (3D) printing has emerged as a promising technology for the fabrication of energy devices due to its unique capability of manufacturing complex shapes across different length scales. 3D-printed energy devices can have intricate 3D structures for significant performance enhancement, which are otherwise impossible to achieve through conventional manufacturing methods. Furthermore, recent progress has witnessed that 3D-printed energy devices with micro-lattice structures surpass their bulk counterparts in terms of mechanical properties as well as electrical performances. While existing literature focuses mostly on specific aspects of individual printed energy devices, a brief overview collectively covering the wide landscape of energy applications is lacking. This review provides a concise summary of recent advancements of 3D-printed energy devices. We classify these devices into three functional categories; generation, conversion, and storage of energy, offering insight on the recent progress within each category. Furthermore, current challenges and future prospects associated with 3D-printed energy devices are discussed, emphasizing their potential to advance sustainable energy solutions.

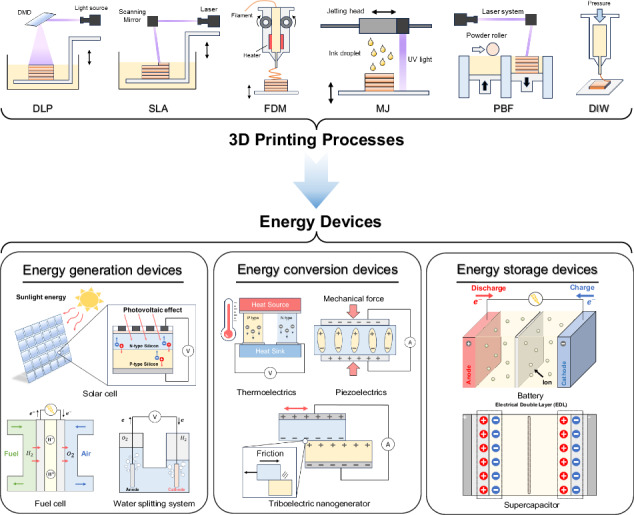

## Introduction

Despite their detrimental environmental effects, fossil fuels continue to dominate global energy consumption, contributing to approximately 70% of the world’s ${{\rm{CO}}}_{2}$ emissions^1,2^. In response to these environmental challenges, researchers have been exploring renewable and carbon-neutral energy sources to reduce our dependence on fossil fuels^3,4^. To achieve these goals, significant efforts have been recently dedicated to developing next-generation energy devices that can meet the ever-growing demand for energy in a more sustainable and environmentally friendly manner^5,6^. State-of-the-art energy devices can be classified into three main groups based on their functions: energy generation, energy conversion, and energy storage^7–9^. Energy generation devices, such as solar cells, fuel cells, and water splitting systems, have the capacity to generate electricity from ambient environments or other energy sources. Energy conversion devices, including piezoelectric devices, triboelectric nanogenerators, and thermoelectric generators, convert the energy harvested from the surrounding environment into usable electrical energy. Lastly, energy storage devices, such as supercapacitors and batteries, enable the storage and release of energy in an electrochemical manner, facilitating efficient energy utilization and management.

Traditional manufacturing methods for the production of energy devices and their parts include melt spinning, injection molding, solution casting, electrospinning, spin coating, sputtering, electrochemical deposition, and chemical vapor deposition^10,11^. However, these conventional techniques suffer from process complexity, extended production times, high costs, and low material utilization rates due to their subtractive nature. Moreover, with the growing demand for wearable/flexible energy devices, conventional fabrication processes present obstacles in achieving miniaturization and customization of these devices^12^. Furthermore, in electrochemical energy devices such as water splitting systems, supercapacitors, and batteries, precise dimensional control and the intricate three-dimensional structure of electrodes play a crucial role because these factors directly impact the mechanical properties and electrical output performances of the energy device^13,14^. For example, the energy density and areal capacitance of electrochemical energy devices increases with the electrode thickness. Additionally, an electrode featuring a microcellular porous structure demonstrates higher power/energy density due to the increased surface area. As the existing production methods exhibit limited controllability over these parameters, alternative manufacturing methods are being explored to enhance the mechanical and electrical output performances of energy devices.

In recent years, three-dimensional (3D) printing, also formally known as additive manufacturing (AM), has been spotlighted as a promising technology for fabricating energy devices due to its unique characteristics, including rapid prototyping, customizability, material diversity, process flexibility, and precise geometry controllability^15–17^. 3D printing enables the formation of 3D objects by directly combining constituent materials, often layer by layer, following pre-designed 3D computer models. Through this approach, energy devices with highly intricate structures such as hierarchically porous micro-lattice designs can be fabricated, which is unattainable through conventional manufacturing methods^18–20^. Furthermore, several studies have demonstrated the 3D printing of energy devices with microcellular architectures that exhibit notable attributes such as high surface area and high stiffness-to-weight ratio. This approach has yielded the improvements in both electrical output performances (specific capacity, energy density, power density, etc.) and mechanical properties (flexibility, deformability, strength, toughness, stiffness, etc.) compared to their bulk counterparts^21–23^.

Among the diverse array of 3D printing processes^16,24,25^, fused deposition modeling (FDM), direct ink writing (DIW), powder bed fusion (PBF), stereolithography (SLA), digital light processing (DLP), and material jetting (MJ) methods have been predominantly utilized for the fabrication of energy devices. In FDM, a thermoplastic polymer is continuously fed as a filament into a heated nozzle, where it is melted and extruded in three dimensions to create the desired object. Subsequent cooling and solidification result in the final 3D structure. In DIW, a viscous material is extruded through a nozzle that moves according to a pre-programmed path in three-dimensional space, constructing a 3D object. PBF employs a high-power laser beam as an energy source that scans over a thin layer of uniformly spread particles to selectively melt and fuse the particles together into a 3D object. SLA and DLP methods both harness light energy to transform a liquid photo-curable polymer into a solid object within a vat. SLA involves the precise scanning of a laser beam over a photo-curable resin, solidifying it point by point, whereas DLP utilizes a digital dynamic mask to project a 2D UV light pattern, solidifying an entire layer all at once. Finally, MJ is a 3D printing technique that involves the precise deposition of building material. In this process, a printhead dispenses numerous tiny droplets of a photocurable substance, which are subsequently solidified through UV irradiation.

**Fig. 1:**
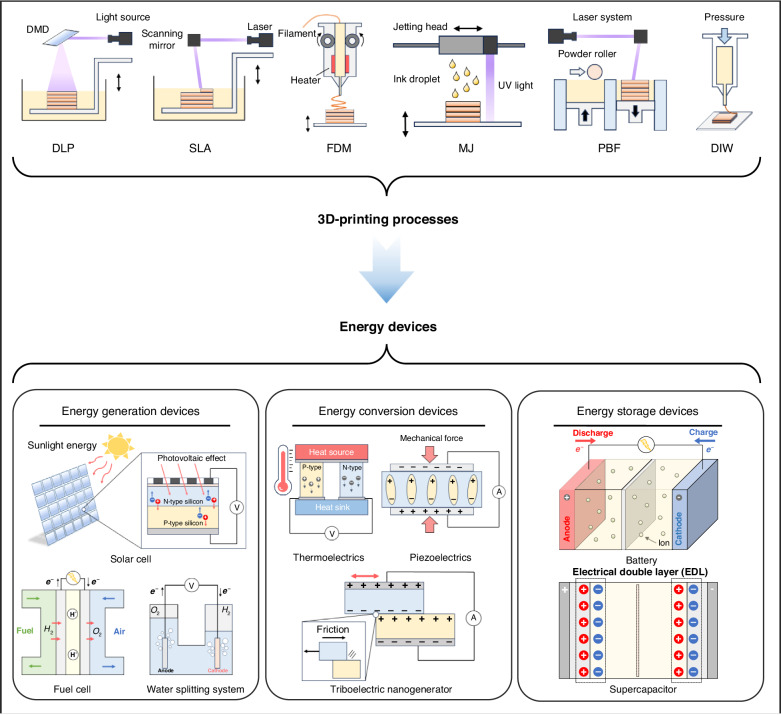
Overview of 3D printed energy devices: from various 3D printing processes (Digital light processing (DLP), Stereolithography (SLA), Fused deposition modeling (FDM), Material jetting (MJ), Powder bed fusion (PBF), Direct ink writing (DIW)) to 3D-printed energy devices (energy generation devices (solar cell, water splitting system, fuel cell), energy conversion devices (triboelectric nanogenerator, piezoelectrics, thermoelectrics), and energy storage devices (battery, supercapacitor))

Despite many recent publications, including review articles, focusing on 3D printing of energy applications^14,26–29^, there remains a notable absence of a concise review that collectively covers the wide spectrum of energy life cycle, from generation and conversion to storage, within the realm of 3D printing. This review aims to fill this gap by providing a brief overview of recent progress in 3D-printed energy devices across diverse energy applications. We organize the state-of-the-art 3D-printed energy devices into three main categories of energy generation devices, energy conversion devices, and energy storage devices, and present an overview of significant developments within each category (Fig. 1). Finally, we briefly discuss the current challenges and prospects associated with 3D-printed energy devices.

## 3D printed energy generation devices

### Fuel cell

**Fig. 2:**
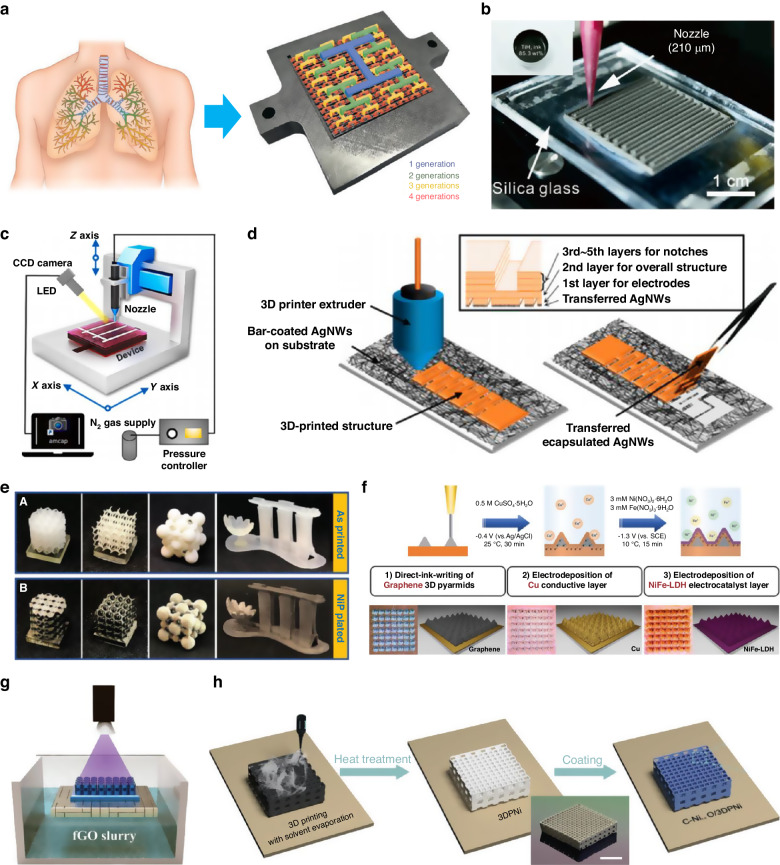
3D printed energy generation devices. **a** Lung-inspired flow fields for PEFCs. Reproduced with permission from Royal Society of Chemistry (2018)^30^. **b** Top view and detail of the cross-section of the self-standing 3D printed 8YSZ membranes. Reproduced with permission from Elsevier (2021)^31^. **c** Schematic of the DIW printing setup for a-ITO/Ag mesh TCE printing. Reproduced with permission from Elsevier (2022)^43^. **d** Schematic of the 3D printed kirigami/origami with silver nanowires (AgNWs). Reproduced with permission from American Chemical Society (2019)^45^. **e** 3D printed polymeric objects and their NiP plated counterparts. Reproduced with permission from Wiley (2019)^49^. **f** Schematic illustration of the fabrication of 3D printed pyramid electrodes for OER in three successive steps. Reproduced with permission from Springer Nature (2022)^50^. **g** Schematic of the fabrication of graphene-based capillary array. Reproduced with permission from Wiley (2023)^52^. **h** Schematic diagrams showing the preparation of 3DPNi and the surface functionalization of 3DPNi with C-Ni1-xO/3DPNi lattice structure. Reproduced with permission from Wiley (2020)^53^

Fuel cells (FCs) are devices that generate electrical energy through the electrochemical reaction of a fuel and an oxidizer. Due to their utilization of hydrogen as a clean fuel source and their resulting production of pure water as the sole byproduct, FCs are emerging as a promising technology for sustainable future. Conventional manufacturing techniques, such as screen printing, dry pressing, chemical vapor deposition, and spray painting, are employed in the fabrication of FCs. However, these current methods have limitations such as limited resolution, suboptimal mechanical properties, elevated costs, increased material usage, and constraints in producing intricate microstructures. 3D printing holds the potential to overcome these limitations and to contribute to the production of more energy-efficient FCs. For example, as shown in Fig. 2a, a lung-inspired flow-field design was introduced to manage non-uniform reactant transportation issues in polymer electrolyte fuel cells (PEFCs)^30^. A lung-inspired structure, printed by FDM, showed uniform gas distribution and a lower pressure drop than the flow field of a conventional serpentine structure. This led to an even distribution of reactants and an approximately 30% increase in power density. Similarly, in PEFC, DIW was employed to integrate the flow field and the gas diffusion layer (GDL), as shown in Fig. 2b^31^. By employing a porous bone-like structure of Ti, water transport was accelerated through capillary action, while regularly printed gaps facilitated the transport of gases. As a result, the new design exhibited a 15% increase in peak power density (PPD). In addition, 3D printed intricate structures can also help increase the active area at the electrodes. For example, corrugated 8 mol% yttria-stabilized zirconia electrolytes was fabricated, resulting in the increase in the active area of electrolyte of solid oxide fuel cells (SOFCs) by approximately 60% compared to their planar counterparts^32^. Notably, the maximum power density of corrugated cells has improved by 57%, which is straightforwardly proportional to the enlargement in active area compared to planar cells. Moreover, 3D printing can also fabricate porous carbon structures for use as microbial fuel cells (MFCs) anodes, and by employing carbonization processes, a multiscale 3D porous carbon structure was achieved^33^. These 3D porous carbon structures resulted in a maximum output voltage of 453.4 ± 6.5 mV, an open-circuit potential of 1256 ± 69.9 mV, and a power density of 233.5 ± 11.6 mW m^−2^. Another advantage of using 3D printing is the ability to leverage inherent manufacturing characteristics. For example, DLP was utilized to produce electrodes for SOFCs in batches, leveraging the intrinsic capability of DLP to arrange multiple geometries within a single layer simultaneously^34^. In addition, additive nature of 3D printing can help to reduce material waste, which, in turn, results in cost reduction during mass production. For example, an MFC in which all components are printed using commercial filament was reported^35^. Although the raw material costs are slightly higher than those of the conventional cation exchange membrane (CEM), it has been suggested that these costs could significantly decrease with mass production. Another example was the use of 3D-printed thermosetting reusable cells in the fabrication of a microfluidic photo fuel cell (µpFC)^36^. Conventional µpFCs are typically made entirely using PDMS, which is not reusable. However, FDM can be used to fabricate reusable cells and templates for PDMS curing, significantly reducing the amount of material used. In addition to the previously mentioned cases, open-source 3D printers can be adapted to enable the creation of new printing systems on demand. For instance, a commercially available 3D printer was modified to manufacture electrodes for PEMFCs^37^. 3D printers using ceramics are rarely affordable due to their expensive costs, so a cost-effective FDM printer was modified and combined with a micro-extrusion system for ceramic materials. Moreover, a DIW system with a temperature controller attached to the syringe has been reported^38^. Since the paraffin-based slurry, consisting of Ce_0.8_Sm_0.2_O_1.9_ (SDC) and sodium cobaltite (SC) powders, needs to remain at 90 °C, a thermostat was used to control the temperature.

### Solar cell

Solar cells (SCs) directly convert solar energy into electrical power without the use of fossil fuel, making them highly valuable as a sustainable energy solution. SCs are divided into three generations based on their stage of commercialization: first-generation crystalline silicon, second-generation thin-film silicon and copper indium gallium selenide (CIGS), cadmium telluride (CdTe), and third-generation dye-sensitized solar cells (DSSC), organic solar cells (OPV), and perovskite^39^. SCs are fabricated with several functional layers (e.g. photoactive layer, transport layer, buffer layer and back electrode layer), and each layer is closely connected, so facile and reliable fabrication methods are essential^40^. Mainstream methods such as spin coating, screen printing, scraping, and gravure printing have significant drawbacks, including uneven layer thickness and low uniformity. In this regard, 3D printing can be considered as a candidate for future SCs manufacturing due to its high resolution, fabrication accuracy, and the ability to reconfigure the system as needed. For example, slot-die coating methods, which modify commercial FDM printers, were introduced to manufacture roll-to-roll SCs^41^. Most printing methods for SCs initially produce a wet film and then allow it to dry naturally or with hot air, which is slow and can lead to poor material uniformity within the film. To avoid this, a temperature-controlled substrate was used to quickly dry the solvent, and a “gas quenching” method, involving the spraying of nitrogen gas, was used to prevent excessive crystallization of the film solution. Consequently, the power conversion efficiency (PCE) of fully printed perovskite SCs reached 11.96%. Similarly, in polymer solar cells (PSCs), FDM-based slot-die coating was used to analyze the optimal temperature of hot solution and hot substrate^42^. In PSCs, the nano-morphologies, molecular orientation, and charge recombination of the active film changes with temperature. Therefore, the optimal temperature was investigated by varying the slot head temperature (solution temperature) and the temperature of the substrate, resulting in a PCE of 7.06% at 80 °C–80 °C respectively. In addition, 3D printing can be employed for the fabrication of semitransparent perovskite SCs (ST-PSCs). To enhance the performance of ST-PSCs, it is crucial to deposit a transparent conducting electrode (TCE) without damaging the fragile PSC surface. Using DIW, an Ag mesh was printed as a TCE on an amorphous indium-tin-oxide (a-ITO) film with high precision (Fig. 2c), resulting in 85% transmittance in the near-infrared region and a 16.26% power conversion efficiency (PCE)^43^. Additionally, 3D printing can be employed for the rapid and straightforward fabrication of the intricate structures that constitute a solar cell. For instance, inspired by the vein network of a leaf, a novel morphology for the counter electrodes (CEs) in DSSCs has been reported^44^. This fractal-based design resulted in a significant increase in the active area compared to conventional planar designs, enhancing catalytic activity and ultimately leading to improved performance. Another example involved the use of FDM for the printing of origami-based photovoltaic electronics that are both stretchable and possess high areal coverage (Fig. 2d)^45^. It was demonstrated that highly elastic thermoplastic polyurethane (TPU) materials could be printed using FDM and combined with highly electrically conductive AgNWs, resulting in a 400% system stretchability and 25,000% interconnect stretchability, making them suitable for application in wearable devices.

### Water splitting system

Water splitting (WS) is an electrochemical technology that electrolyzes water to separate ions through a membrane, generating hydrogen and oxygen. A typical electrochemical WS system includes two key reactions: the oxygen evolution reaction (OER) and the hydrogen evolution reaction (HER). Evaluating the performance of the electrode in these reactions depends on various parameters, such as the conductivity of the catalyst, the support material responsible for charge transfer, surface geometry, and the effective catalyst area^46^. In this regard, the advent of 3D printing has been a breakthrough in the field of WS by allowing for greater design freedom and the use of a variety of materials and simple processes. For example, 3D-printed stainless steel with electrodeposition procedure was reported^47^. By using SLM 3D printing, a variety of designs for electrodes were achieved with subsequent electrodeposition that compensated for the disadvantages of pure stainless-steel electrodes, resulting in the enhancement of their electrocatalytic properties toward hydrogen and oxygen evolution. In addition, SLM was also used for the fabrication of 3D printed conical arrays of TiO_2_ which split water to produce hydrogen under UV illumination^48^. While focused ion beam lithography has been a typical fabrication method for Ti, it is slow and difficult to scale to large substrates. SLM 3D printing was thus utilized to achieve three dimensionally patterned photocatalytic Ti surface with high throughput and large scale compared to the point-by-point ion beam lithography method. Furthermore, mechanical metamaterial electrodes for WS have been reported, as shown in Fig. 2e^49^. These electrodes possess a flexible structure with high conductivity, owing to an amorphous nickel-phosphorous coating that demonstrates a low resistivity of 0.45 µΩ m at 298 K, making them well-suited for WS applications. Another example involves the use of DIW to print a 3D pyramid electrode for the OER (Fig. 2f)^50^. This electrode is composed of functional graphene ink, and it underwent successive electrodeposition of Cu and NiFe-layered double hydroxide (NiFe-LDH). The introduction of these 3D pyramid structures led to an expansion in the electrode’s surface area and active sites, resulting in an enhancement of the OER activity. In addition to the aforementioned advantages, by using 3D printing, mechanically robust and functionalized structures were fabricated. For example, mechanically robust graphene/carbon nanotubes (CNT) electrodes, which utilize 1D CNT to reinforce friction and adhesion between 2D graphene nanosheets, was reported^51^. Previous 3D printed graphene electrodes were susceptible to failure under common circumstances such as surface traction and buoyancy. However, by incorporating 1D CNTs between the 2D graphene nanosheets, increased flexural stiffness effectively addressed this issue. Additionally, bio-inspired catalyst support inspired by water transport mechanisms in capillary plants was reported^52^. A graphene-based capillary array was fabricated as a catalyst support using DLP (Fig. 2g), and highly active CoNi carbonate hydroxide (CoNiCH) was subsequently deposited on this support to facilitate water supply and bubble release. Furthermore, 3D printed Ni electrodes (3DP Ni) were manufactured using both DIW and DLP techniques. Conventional Ni electrodes often suffer from low current densities due to the formation of bubbles and stochastic porous structures. To address these issues, DIW (Fig. 2h)^53^ and DLP^54^ was used to create 3DP Ni electrodes with a periodic pore structure. The 3DP porous Ni electrodes promotes bubble migration, enhancing the accessibility of catalytic active sites. Furthermore, the large active area inherent in conventional Ni electrodes, due to the micropore structure, is harnessed to achieve high current densities.

## 3D printed energy conversion devices

### Piezoelectric device

**Fig. 3:**
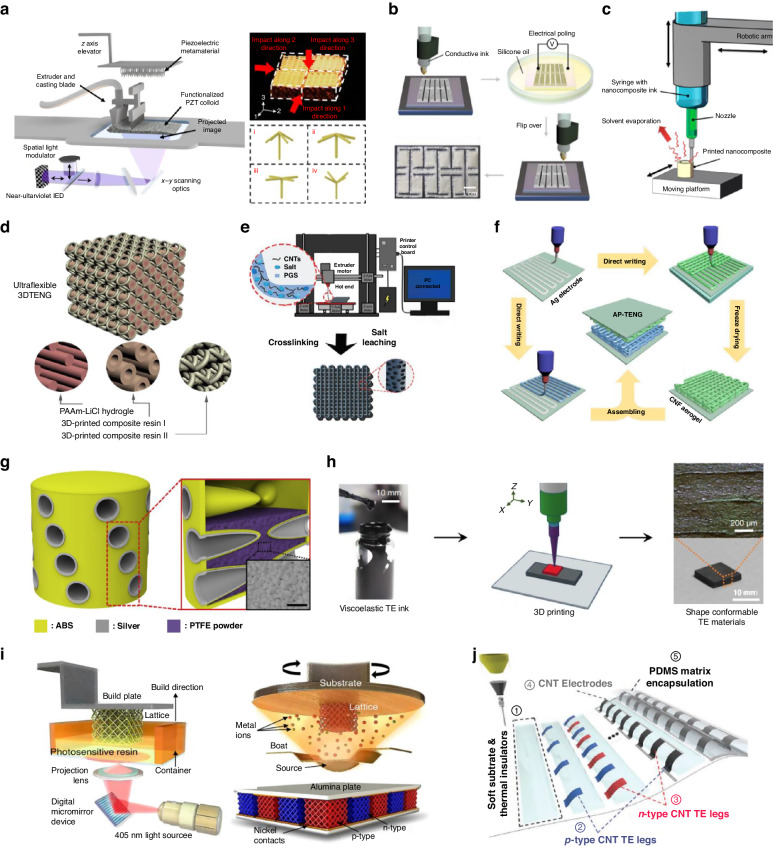
3D printed energy conversion devices. **a** High-resolution DLP 3D printing of piezoelectric metamaterials with encoded anisotropic piezoelectric constants. Reproduced with permission from Springer Nature (2018)^57^. **b** Fabrication process of the printed piezoelectric device with kirigami structure. Reproduced with permission from Elsevier (2020)^59^. **c** One step solvent evaporation-assisted 3D printing process. Reproduced with permission from American Chemical Society (2017)^61^. **d** 3D printed ultraflexible TENG with shell structure. Reproduced with permission from Elsevier (2018)^70^. **e** Fabrication of the 3D printed TENG with hierarchical porous design. Reproduced with permission from Wiley (2018)^71^. **f** 3D printed biomimetic villus structure with enhanced surface area. Reproduced with permission from Elsevier (2019)^72^. **g** 3D hierarchically patterned TENG printed by DIW. Reproduced with permission from Elsevier (2019)^73^. **h** 3D printing of shape-conformable TE materials using viscoelastic all inorganic TE inks. Reproduced with permission from Springer Nature (2019)^81^. **i** DLP printing of architected thermoelectric microlattices with exceptional mechanical and electrical properties. Reproduced with permission from Springer Nature (2023)^84^. **j** All-printed 3D-compliant TEGs with notable mechanical reliability and electrical output through DIW process. Reproduced with permission from Wiley (2023)^85^

Piezoelectric materials, which can convert mechanical energy into electrical energy and vice versa, have found extensive use in various fields such as sensing, actuation, energy harvesting, ultrasonic transduction, and microelectromechanical systems (MEMS)^55,56^. Nowadays, 3D printing has emerged as a highly promising advanced manufacturing process for producing piezoelectric devices with complex three-dimensional designs and tunable properties across multiple scales for various applications. For instance, PZT nanocomposites with intricate 3D architectures and arbitrary piezoelectric coefficient tensors was successfully fabricated through high-resolution DLP printing (Fig. 3a)^57^. In contrast to conventional piezoelectric materials, where the piezoelectric charge constant is determined by the inherent crystal structure of the constituent materials, this work introduced an approach to modify the piezoelectric coefficient by arranging a set of piezoelectric architectural units in specific spatial patterns. Also, four unique types of piezoelectric lattice cells—standard, cross, sandwich, and pyramid—were created using FDM 3D printing^58^. These lattice cells possessed varying internal density distributions and were capable of regulating piezoelectric performance without altering the material composition or the physical geometry (shape, size, and mass) of the piezoelectric devices. Furthermore, 3D printing of a specially designed kirigami structure comprising BaTiO3/P(VDF-TrFE) composite and a silver-based 3D printed electrode was realized through DIW printing (Fig. 3b)^59^. This innovation overcame the issues associated with common stretchable kirigami structures, resulting in a stretchable piezoelectric device suitable for pressure-based modes. Meanwhile, the molecular dipoles within most piezoelectric materials are randomly oriented within their crystal structures. Achieving an effective piezoelectric response from these materials requires an electrical poling process, which entails additional time post 3D printing and necessitates the use of equipment with a high-voltage source. To streamline this process, electric poling-assisted 3D printing has been developed, which combines electrical polarization with fused deposition, allowing simultaneous polarization and printing to create piezoelectric devices of any desired shape^60^. Furthermore, several efforts have been made to mechanically align the molecular dipoles of piezoelectric materials during the 3D printing process, eliminating the need for additional electrical poling. For instance, a one-step solvent evaporation-assisted 3D printing technique (Fig. 3c) was developed, and a nanocomposite formulation was engineered to achieve piezoelectric properties and printability comparable to commercial PVDF films without the necessity of electrical poling^61^. Similarly, ionic liquid (IL)-assisted FDM methods was introduced for the direct printing of β-PVDF piezoelectric devices^62^. The shearing force imparted by FDM aided in the directional alignment of dipoles, resulting in self-polarization characteristics in the printed PVDF devices without the requirement for electrical poling. Furthermore, due to their exceptional attributes including unprecedented functionality, customizability, and high sensitivity, 3D printed piezoelectric devices have been applied across a range of practical applications, including airflow sensing, robotic metamaterials, ultrasonic transducers, and impact sensors^63–65^.

### Triboelectric nanogenerator

Triboelectric nanogenerators (TENGs), first introduced by Z.L. Wang’s group in 2012, convert ambient mechanical energy into electricity through the synergistic mechanisms of contact electrification and electrostatic induction^66^. The intrinsic merits of TENGs, including their broad material availability, adaptability, utilization of ubiquitous resources, and remarkable efficiency, have led to their extensive exploration and implementation across diverse domains, encompassing self-powered sensors, actuators, robotics, wearables, and even implantable devices^67–69^. Recent advancements have seen the integration of various 3D printing methodologies in the fabrication of TENGs. This innovative methodology has enabled the creation of 3D printed TENGs endowed with distinctive structural designs and heightened functionalities, unattainable through conventional fabrication techniques. For example, a pioneering shell-structured ultra-flexible triboelectric energy-harvesting device shown in Fig. 3d was introduced as the first 3D-printed TENG^70^. By utilizing a composite resin and a hydrogel as triboelectric materials in conjunction with an electrode, they achieved a layered columnar array configuration facilitated by the precision of 3D printing. Additionally, an elastic and integrated 3D printed TENG featuring a hierarchical porous structure was developed^71^. This structure was seamlessly fabricated through a one-step DIW process (Fig. 3e), eliminating the need for supplementary assembly. Each discrete pore functions as an individual TENG unit, where the composite carbon nanotubes (CNTs) and the poly(glycerol sebacate) (PGS) matrix serve as the triboelectric components. Simultaneously, the interconnected CNTs establish a conductive network serving as electrodes. Meanwhile, several recent endeavors have focused on leveraging 3D printing technology to improve the energy conversion efficiency of TENGs. A high-performance biocompatible TENG based on cellulose, boasting a hierarchically patterned 3D micro/nano structure, was fabricated via DIW printing (Fig. 3f)^72^. This intricate design significantly augmented the utilization of the structure, contributing to enhanced contact area, surface roughness, and mechanical resilience. As a result, the triboelectric response was substantially improved, with the 3D printed TENG generating nearly 175% more voltage output compared to its traditionally molded counterpart. Likewise, an innovative ABS-based biomimetic villus structure (Fig. 3g) was printed through DLP printing, significantly augmenting the triboelectric surface area in contact with polytetrafluoroethylene (PTFE) powder^73^. This innovation led to a 4–5 folds enhancement in power output. Moreover, the efficacy of printed structures with elastic supporting substrates was demonstrated, utilizing PTFE as the triboelectric material and copper film as the electrode on a distinct surface of the framework^74^. These 3D printed structures effectively increased the number of triboelectric contact-separation pairs, amplifying the active surface contact area within a single device and consequently advancing rehabilitation capabilities. In addition to aforementioned cases, capitalizing on its customizability, unparalleled versatility and diverse material compatibility, 3D printed TENGs has been utilized in contemporary applications, encompassing various targeted domains including noise cancellation, self-healing materials, dust filtration, and physical condition monitoring^71,73,75,76^.

### Thermoelectric generator

Thermoelectric generators (TEGs) can directly convert heat into electricity and vice versa based on the Seebeck and Peltier effects of thermoelectric materials. TEGs have attracted considerable interest owing to their distinctive advantages including environmental friendliness, scalability, reliability, and silent operation^77,78^. The output power of TEGs hinges on the heat transferred from heat sources and the energy conversion efficiency of the thermoelectric (TE) materials^79^. However, while TE materials have witnessed improvements in energy efficiency, conventional TEG geometries, primarily restricted to a cuboid or other symmetrical shapes, have led to suboptimal power outputs due to inefficient thermal contacts with curved heat sources such as exhaust pipes or the human body^80^. In this regard, recently, several efforts have been devoted to fabricating TEGs using novel 3D printing technologies, aiming to establish conformal interfaces on three-dimensional heat sources of arbitrary shapes. An example was reported where an extrusion-based 3D printing method was employed for printing ${{Bi}}_{2}{{Te}}_{3}$-based TE materials (Fig. 3h)^81^. This work used all-inorganic colloid inks with ${{Sb}}_{2}{{Te}}_{3}$ chalcogenidometallate (ChaM) ions as inorganic binders. This approach enabled the production of TE materials conforming to heat sources of any shape. ChaM ions facilitated moderate viscoelasticity in colloidal inks and enabled layer-wise deposition of 3D TE structures without compromising TE performance as often occurs with organic binders. They further refined this process in a subsequent paper, achieving finer control over 3D printing resolution, enabling fabrication of microscale three-dimensional TE architectures with dimensions below 200 µm^82^. Optimization of particle size, size distribution, and surface oxidation of ink materials facilitated extremely high viscoelasticity of the inorganic thermoelectric inks. Moreover, novel methods for integrating 3D printing into TEG production was introduced, emphasizing shape versatility^83^. They utilized SLA printing to create scaffold structures (cuboid, cylindrical gear, sawtooth shape), coated these scaffolds with commercially available p-type PEDOT and self-formulated n-type ${{Ag}}_{2}{Se}$, and ultimately manufactured TEGs with varied targeted shapes. In addition, for enhanced TEG energy conversion efficiency, printable TE materials should concurrently exhibit outstanding TE properties and mechanical stability against deformation for conformal contact with diverse heat sources. For instance, a solution to key limitations in 3D printed TEGs, brittleness and low power efficiency, was reported as shown in Fig. 3i^84^. The authors designed core–shell TE 3D micro-lattice architectures comprising hybrid carbon cores and TE shells (p-${{Sb}}_{2}{{Te}}_{3}$ and n-${{Bi}}_{2}{{Te}}_{3}$). Leveraging the synergy between the robust, ductile core and low-dimensional shell, they achieved exceptional specific toughness and remarkable power conversion efficiency, surpassing previously reported monolithic TEGs. Similarly, integrated 3D-compliant TEGs (Fig. 3j), entirely 3D printed through DIW using pre-doped CNT inks and polydimethylsiloxane (PDMS), displayed the highest normalized open-circuit voltage (0.28 *mV K*^*−1*^ cm^*−2*^) among the additively manufactured TEGs and notable mechanical reliability against repetitive deformation^85^. Recent research has also demonstrated that 3D printed TEGs can possess unique mechanical properties and functionalities unattainable through conventional manufacturing methods. For example, a TE material with high stretchability and rapid self-healing ability was demonstrated through 3D printing^86^. Moreover, thermal explosion and SLM printing was combined to generate highly textured bulk TE materials, in which the printed TE materials had a high preferential orientation factor of 0.9 and displayed up to a 195% increase in compressive strength (91 MPa) compared to conventional zone-melted samples^87^.

## 3D printed energy storage devices

### Battery

**Fig. 4:**
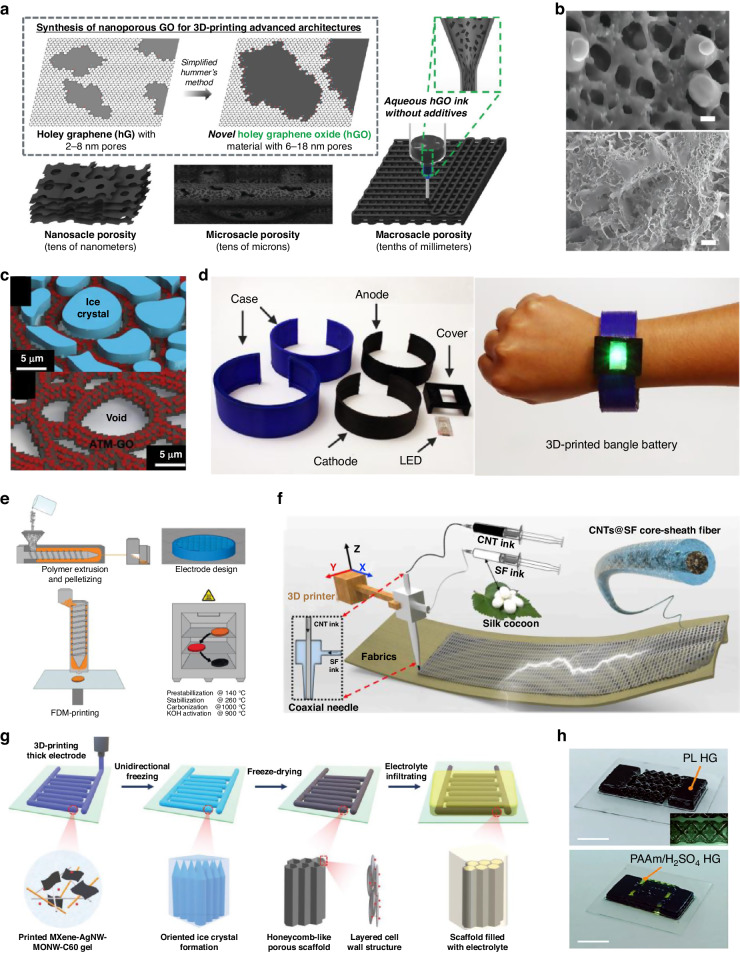
3D printed energy storage devices. **a** Schematic illustration of synthesize process and nano-, micro-, and macro-porosity. Reproduced with permission from Wiley (2018)^90^. **b** SEM images of a composite electrode without dendrite growth (top) and a pure Zn electrode having dendrite growth (bottom). Reproduced with permission from Wiley (2023)^91^. **c** Schematic illustration of macrostructure in aerogel after freeze drying (top) and aerogel after free-drying (bottom). Reproduced with permission from Elsevier (2019)^96^. **d** 3D printed battery serving as a structural element. Reproduced with permission from American Chemical Society (2018)^97^. **e** Fabrication process of the 3D printed supercapacior using polymer EAM and annealing. Reproduced with permission from Wiley (2022)^100^. **f** 3D printing of textiles using CNTs and CMC by coaxial spinneret. Reproduced with permission from Elsevier (2019)^101^. **g** Schematic diagrams of the fabrication of a honeycomb structure with DIW and unidirectional freezing. Reproduced with permission from Wiley (2020)^103^. **h** Micro-supercapacitor based on 3D printed octet-truss design. Reproduced with permission from Wiley (2023)^104^

In batteries, electrical energy is generated by converting chemical energy through redox reactions at the anode and cathode, where the anode serves as the negative electrode and the cathode as the positive electrode, collectively enabling the energy storage process. Batteries had been commercialized in various field and appliances from smartphone to electric vehicles recently, owing to their advantages such as untethered energy supply, portability, long lifespan, rechargeability, and low maintenance costs^88,89^. Recently, 3D printing has enabled improved performance, lifespan, and fixed problem of batteries which cannot be applied in current fabrication method. For instance, leveraging 3D printing, perforated scaffolds were meticulously engineered across nano-, micro-, and macroscopic scales (Fig. 4a). This innovative approach yielded a remarkable upsurge in battery performance for the 3D-printed ${\rm{Li}}-{{\rm{O}}}_{2}$ cathode-exhibiting an astounding 42-fold enhancement in comparison to the conventional 2D vacuum-filtered film configuration^90^. In other case, 3D printing creates a self-healing, highly conductive, porous structure, which enables cyclic plating and stripping of Zn-ions because there is no Zn dendrite growth and deformation (Fig. 4b)^91^. Another issue is shuttling effect which occurs due to dissolution of polysulfide in organic electrolyte and causes degradation of cycling performance. Microscale pores was printed through DIW to offer the capacity to secure polysulfide species onto the cathode, effectively alleviating the detrimental shuttling effect and thereby fostering enhancements in cycling performance^92,93^. In contrast to conventional bulk electrodes, the lattice battery electrodes demonstrates enhanced performance, further augmented by the sintering procedure that imparts a porous configuration^94^. This causes an elevated surface-to-volume ratio, thereby endowing the lattice with a charge capacity exceeding twofold that of the conventional solid bulk electrode. Furthermore, the lattice configuration was incorporated using DLP to produce carbon electrode having reduced density and heightened strength without the inclusion of binders or conductive additives^95^. Electrode was yielded by exemplifying both exceptional compressive strength and elevated ionic mobility while inherently retains its structural integrity. In some cases, the integration of separate approaches during or after 3D printing has resulted in improvements to existing issues or performance gains. For example, inkjet printing was combined with freeze casting, thereby inducing the formation of ice microcrystals during the printing process to precisely regulate both microstructure and microporosity (Fig. 4c)^96^. By means of freeze drying, executed at low temperatures, and subsequent thermal annealing, the ${\rm{Mo}}{{\rm{S}}}_{2}$ precursor and GO transform into a hybrid configuration, seamlessly adhering to the surface of the profoundly porous rGO framework. This hybrid construct significantly bolsters electrical conductivity and mechanical resilience, all the while expediting ion transport pathways. In addition, the freedom of 3D printing in shape design has been utilized to change the form factor of batteries from plane geometries to conformal structural designs as shown in Fig. 4d^97^. The enhancement of the mechanical property of a battery was also demonstrated by printing it with a robust polymer^97,98^.

### Supercapacitor

Supercapacitors (SC) operates by utilizing the alignment of electrolyte ions at the interface between the electrolyte and the electrode, forming electrical double layers (EDLs) that store and release energy. This results in the parallel movement of electrons through the external wire. SCs offer advantages such as long lifespan, high power density and rapid charging capability within short timeframes, making them well-suited for diverse applications including memory protection at electric circuit and electric vehicle^88,99^. However, SCs also have limitations such as self-discharge which lowers SC’s power-delivery ability. 3D printing has recently been employed to overcome this limitation. Specifically, 3D printing of Polyacrylonitrile (PAN) polymer allows for the direct production of carbon electrodes with diverse shapes (Fig. 4e), eliminating the need for solvents in the process that imposes constraints for freeform fabrication^100^. By leveraging polymer extrusion-based techniques with annealing for pre-stabilization, the proposed approach allows for the fabrication of tailored 3D electrode structures with enhanced performance in SCs. In addition, DIW printing was used to fabricate smart textiles composed of carbon nanotubes (CNTs) and carboxymethyl cellulose (CMC) in a layer-by-layer fashion, as shown in Fig. 4f^101^. CMC was incorporated as a solid electrolyte and flexible layer to enhance the resistance to mechanical stress. This smart textile has the capability to harvest biomechanical energy from human movements and achieve a high-power density of 18mW m^−2^. Additionally, using DIW, a quasi-solid state micro-supercapacitor was successfully manufactured, resulting in a remarkable energy density of 73.9 ${\rm{\mu }}$Wh/cm^2,102^. This novel advancement not only facilitated simplified assembly onto both cathode and anode compared to traditional micro-supercapacitors, but also demonstrated enhanced mass transportation within the device due to the 3D printed porous electrodes. Furthermore, through the strategic integration of DIW with the concept of unidirectional freezing, micro superconductors (MSCs) with enhance performance have been engineered successfully (Fig. 4g)^103^. This approach involves the formation of a porous architecture reminiscent of a honeycomb structure, which effectively mitigates the inherent challenge of low areal capacitance observed in traditional MSCs. Recently, an octet-truss structure electrode shown in Fig. 4h has been produced by DLP technology and incorporated in MSCs^104^. This innovation distinctly enhanced the interaction interface between the electrode and the electrolyte, resulting in a consequential augmentation of ion absorption on the electrode surface. While having augmented weight or increased electrode thickness, capacitive materials suffer from the limited capacitance. To address this shortcoming, the weight of ${\rm{Mn}}{{\rm{O}}}_{2}$, a frequently employed capacitive material, was optimized through the implementation of an aerogel scaffold structure after DIW 3D printing^105^. Both Gravimetric capacitances and volumetric capacitances remain same value after ${\rm{Mn}}{{\rm{O}}}_{2}$ loading.

## Conclusion and Outlook

The efficiency of 3D printing technology in manufacturing energy devices has attracted considerable attention, due to notable advantages such as rapid prototyping, customization, diverse material availability, process flexibility, and precise geometry controllability in comparison to traditional manufacturing methods. In this review, we have categorized state-of-the-art 3D-printed energy devices into three sections: energy generation devices, energy conversion devices, and energy storage devices. Furthermore, we have also discussed recent advancements in the significant development of selected devices within each category. 3D printing introduces new possibilities and suggest potential future directions. For energy generation devices, 3D printing facilitates the fabrication of complex 3D structures with high surface-to-volume ratios such as hierarchical and porous structures. This maximizes the active surface area of the device, enhancing its ability to convert electrochemical energy of fuel or external energy sources into electricity. 3D printing also allows researchers to easily incorporate novel materials such as CNTs and graphene, enabling the achievement of superior electrical and mechanical properties. Furthermore, 3D printing provides unprecedented opportunities for the design and production of energy conversion devices with non-planar and complex geometries, commonly encountered in environmental energy sources and within the human body. The ability of 3D printing to manufacture devices with customized designs, facilitating effective conformal interfaces, has the potential to overcome the limitations imposed by traditional manufacturing methods. For energy storage device, utilizing 3D printing provides the flexibility of structural design, enabling the development of batteries and supercapacitors capable of also serving as structural components for weight reduction purposes. Additionally, employing 3D printed electrodes in the form of microlattices can lead to the enhancements in energy storage density by improving the areal capacity and capacitance of batteries and supercapacitors.

The latest 3D-printed energy devices not only facilitate the creation of highly intricate structures with unparalleled resolution, surpassing the capabilities of conventional manufacturing methods, but also holds promise in terms of the performance. Table 1 shows a summary of the output performances of the aforementioned 3D printed energy devices, in comparison with those manufactured using conventional methods^106–130^. While the electrical output performances of the 3D printed energy devices do not always show significant enhancements in comparison to their conventionally manufactured counterparts, 3D printing retains its promise due to its distinctive advantages over traditional methods, including customizability, material diversity, process flexibility, and precise geometric control. Given that the utilization of 3D printing in energy devices fabrication is still in its early stages of research, we anticipate future advancements in device performance of devices through the optimization of printing processes, expansion of printable materials, and exploration of diverse device structures.

**Table 1:** Comparison of the performance of the energy devices fabricated through conventional manufacturing methods and 3D printing technologies

Category	Device type	Fabrication method		Performance	Ref.
Energy generation	Fuel Cell	Conventional	- Casting - Atomic layer deposition - Dip coating	Open circuit voltage (${V}_{{OC}}$) = $0.9 \sim 1.08\,{\rm{V}}$ Peak power density = $240 \sim 415{\rm{m}}\,{\rm{W}}/{{cm}}^{2}$	^106^ ^107^ ^108^
		3D printing	DIW	Open circuit voltage (${V}_{{OC}}$) $=0.98\,{\rm{V}}$ Peak power density $=727\,{\rm{mW}}/{{cm}}^{2}$	^37^
			SLA	Peak power density $=410\,{\rm{mW}}/{{cm}}^{2}$	^32^
			DLP	Open circuit voltage (${V}_{{OC}}$) $=1.04\,{\rm{V}}$ Peak power density $=176\,{\rm{mW}}/{{cm}}^{2}$	^34^
	Solar Cell	Conventional	- Chemical vapor deposition - Atomic layer deposition - Spray pyrolysis	Power conversion efficiency $=12.2 \% \sim 18.6 \%$	^109^ ^110^ ^111^
		3D printing	FDM	Power conversion efficiency $=11.96 \%$	^41^
			DIW	Power conversion efficiency $=26.47 \%$	^43^
			FDM	Power conversion efficiency $=14.90 \%$	^45^
	Water splitting system	Conventional	- Exfoliate - Chemical bath deposition - Annealing	Current density $=10\,{\rm{mA}}/{{cm}}^{2}$	^112^ ^113^ ^114^
		3D printing	DIW	Current density $=30\,{\rm{mA}}/{{cm}}^{2}$	^51^
			DLP	Current density $=500\,{\rm{mA}}/{{cm}}^{2}$	^54^
			DIW	Current density $=600\,{\rm{mA}}/{{cm}}^{2}$	^53^
Energy conversion	Piezoelectric device	Conventional	- Sputtering - Solution casting - Electrospinning	Open circuit voltage (${V}_{{OC}}$) $=0.19 \sim 2\,V$ Sensitivity = $2.82 \sim 22.6\,{mV}/{KPa}$ Piezoelectric charge coefficient (${d}_{33}$) $=31.3{pC}/N$	^115^ ^119^ ^120^ ^121^
		3D printing	DLP	Piezoelectric charge coefficient (${d}_{33}$) $=583{pC}/N$	^64^
			DLP	Piezoelectric charge coefficient (${d}_{33}$) $=38 \sim 110{pC}/N$ Sensitivity = $18.3\,{mV}/{KPa}$	^65^
			FDM	Open circuit voltage (${V}_{{OC}}$) $=4.2V$	^62^
			FDM	Open circuit voltage (${V}_{{OC}}$) $=8.6V$ Sensitivity = $11.87\,{mV}/{KPa}$	^58^
			DIW	Piezoelectric charge coefficient (${d}_{33}$) $=18{pC}/N$ Open circuit voltage (${V}_{{OC}}$) $=4V$	^61^
	Triboelectric nanogenerator	Conventional	- Sputtering - Laser cutting - Spray deposition	Power density (${P}_{D}$) $=1.98 \sim 17.6\,{mW}/{m}^{2}$ Open circuit voltage (${V}_{{OC}}$) $=7.12 \sim 90V$ Closed circuit current$({I}_{{SC}})=0.16 \sim 3.5\,\mu A$	^122^ ^123^ ^124^
		3D printing	DLP	Power density (${P}_{D}$) $=1.4\,{mW}/{m}^{2}$ Open circuit voltage (${V}_{{OC}}$) $=30\,V$	^70^
			FDM	Power density (${P}_{D}$) $=6.7\,W/{m}^{2}$ Open circuit voltage (${V}_{{OC}}$) $=2360\,V$ Closed circuit current$({I}_{{SC}})=1.7\,{mA}$	^74^
			DIW	Power density (${P}_{D}$) $=29\,{mW}/{m}^{2}$ Open circuit voltage (${V}_{{OC}}$) $=55.8\,V$ Closed circuit current$({I}_{{SC}})=940{nA}$	^72^
	Thermoelectric generator	Conventional	- Screen printing - Electrochemical deposition	Figure of merit (ZT) = 0.93 (p-type) Figure of merit (ZT) = 0.64 (n-type) Open circuit voltage (${V}_{{OC}}$) $=68.41\,{mV}$ Power density (${P}_{D}$) $=3 \sim 5230\,\mu W/{{cm}}^{2}$	^116^ ^117^ ^118^
		3D printing	DIW	Figure of merit (ZT) = 1.0 (p-type) Figure of merit (ZT) = 0.5 (n-type) Power density (${P}_{D}$) $=479\,\mu W/{{cm}}^{2}$	^82^
			DIW	Open circuit voltage (${V}_{{OC}}$) $=188.08\,{mV}$	^85^
			DLP	Figure of merit (ZT) = 0.97 (p-type) Figure of merit (ZT) = 1.09 (n-type)	^84^
Energy storage	Battery	Conventional	- Blade casting - Slurry mold - Pellet	Areal capacity =$0.1 \sim 1.53{Ah}/g$ Solid diffusion length $=0.03 \sim 25\,\mu m$	^125^ ^126^ ^127^
		3D printing	DIW	Areal capacity =$0.534\,{Ah}/g$	^93^
			DLP	Solid diffusion length =$8 \sim 30\mu m$	^95^
			SLA	Areal capacity = $3.6\,{Ah}/g$	^98^
			MJ	Areal capacity = $0.43\,{Ah}/g$	^96^
	Supercapacitor	Conventional	- Laser cutting - Mask assisted filtering - Spin coating - Drop casting	Areal capacitance = 0.02$\sim 111.5\,F/{{cm}}^{2}$	^128^ ^129^ ^130^
		3D printing	DIW	Areal capacitance = $207.9\,F/{{cm}}^{2}$	^102^
			DIW	Areal capacitance = $0.2\,F/{{cm}}^{2}$	^103^
			DLP	Areal capacitance = $0.2\,F/{{cm}}^{2}$	^104^
			DIW	Areal capacitance = $44.13\,F/{{cm}}^{2}$	^105^

Despite the aforementioned unique advantages of 3D printing in energy device fabrication, it is also crucial to acknowledge that several challenges must be addressed before achieving widespread commercial adoption of 3D printing in energy applications. First, there are notable limitations in formulating printable inks for 3D printing of energy devices. Table 2 presents a summary of the printing materials associated with various 3D printing techniques reviewed in this article. The printing materials often require the incorporation of crucial additives to ensure printability, such as polymeric binders and rheological modifiers. In most cases, these printing additive materials serve as a matrix to form the 3D geometry, while functional materials, such as CNT, graphene, Ag nanoparticles, PZT, and PVDF, are incorporated in the form of powders or additives to provide energy-related functionalities. However, the inclusion of these additive materials reduces the volume fraction of functional materials, potentially leading to a degradation in the mechanical/electrical performances of the printed devices. For instance, for DIW printing, the inclusion of rheological modifiers is often necessary to impart the specific characteristics such as high viscosity and desired shear-thinning behavior to the printing inks. In the case of SLA and DLP, the printing process requires the electrical material to be suspended in a photocurable precursor solution containing polymers and photo-initiators. FDM relies heavily on thermoplastic polymers that can be melted and extruded at elevated temperatures. The use of these additive and binder materials for printability may unintentionally compromise the electrical and mechanical properties of 3D-printed energy devices. Therefore, substantial efforts are essential to optimize the composition ratios of printing materials for 3D printing energy devices.

**Table 2:** List of materials used for each 3D-printed energy device

Category	Device type	3D printing method	Printing materials	Ref.
Energy generation	Fuel cell	PBF	Stainless steel (Fig. 2a)	^30^
		DIW	TiH2/CH_2_Cl_2_/2-butoxyethanol /dibutylphthalate/PMMA (Fig. 2b)	^31^
	Solar cell	DIW	Silver (Ag) paste (Fig. 2c)	^43^
		FDM	TPU (Fig. 2d)	^45^
	Water splitting system	DLP/SLA	PlasClear/FL01/PlasWhite/RS-F2-GPCL-04 (Fig. 2e)	^49^
		DIW	Graphene microflakes/ethyl cellulose/toluene/xylene (Fig. 2f)	^50^
		DLP	fGO/ACMO/polyester acrylate/TPO (Fig. 2g)	^52^
		DIW	Ni powder/PLGA/DCM/EGBE (Fig. 2h)	^53^
Energy conversion	Piezoelectric device	DLP	PEGDA/PZT (Fig. 3a)	^57^
		DIW	BTO/PVDF/DMF (Fig. 3b)	^59^
		DIW	BTO/PVDF/Acetone/DMF/DMSO (Fig. 3c)	^61^
	Triboelectric nanogenerator	DLP	Liquid photopolymer resin/acrylonitrile butadiene styrene powder (Fig. 3d)	^70^
		FDM	Poly(glycerol sebacate)/Ethyl alcohol/CNTs (Fig. 3e)	^71^
		DIW	Cellulose nanofiber /PDMS/Ag (Fig. 3f)	^72^
		DLP	Commercial photopolymer ABS resin/Ag paste/PTFE powder (Fig. 3g)	^73^
	Thermoelectric generator	DIW	*Bi*_0.4_*Sb*_1.6_*Te*_3_/*Bi*_2_*Sb*_2.7_*Se*_0.3_/ChaM binder (Fig. 3h)	^81^
		DLP	PEGDA/BAPO/*Sb*_2_*Te*_3_/*Bi*_2_*Te*_3_ (Fig. 3i)	^84^
		DIW	Single-walled CNTs/DEG solvent/PAA and PEI additives (Fig. 3j)	^85^
Energy storage	Battery	DIW	Hydrophilic GO (Fig. 4a)	^90^
		DIW	EGaIn/Hemicellulose (Fig. 4b)	^91^
		MJ	Ammonium thiomolybdate/${Mo}{S}_{2}$/GO (Fig. 4c)	^96^
		FDM	LTO/graphene/PLA/LMO/MWNT (Fig. 4d)	^97^
	Supercapacitor	FDM	PAN/Solketal/Acryloyl chloride /Acrylonitrile/DMSO (Fig. 4e)	^100^
		DIW	Silk fibroin Ink/CNT Ink (Fig. 4f)	^101^
		DIW	Mxene/MnONWs/AgNWs/C60 (Fig. 4g)	^103^
		DLP	Acrylamide /PEGDA/TPO/SDS/ PEDOT:PSS (Fig. 4h)	^104^

Second, while we introduced six major 3D printing technologies in this paper (Fig. 1), DIW, DLP, and FDM constitute the majority of the techniques employed (approximately 80% among the works reviewed in this article), whereas MJ, PBF, and SLA are comparatively less utilized. This prevailing trend can be attributed to the accessibility and versatility of the printing methods and materials. FDM, DLP, and DIW 3D printing methods offer greater affordability and ease of access to 3D printers, including the potential for custom-built hardware, in contrast to MJ, PBF, and SLA methods which often necessitate expensive components such as lasers and specialized inkjet heads. Moreover, the simplicity of material requirements (i.e. thermoplastics for FDM, photocurable resins for DLP, shear-thinning viscous materials for DIW) facilitates the development of printable materials with electrical properties necessary for the energy device. In contrast, MJ, PBF, and SLA methods primarily rely heavily on manufacturer-provided commercial printing materials due to the complexity of their systems/processes and stringent material requirements. Looking forward, DIW, DLP, and FDM methods will continue to be extensively utilized in energy device fabrication due to the aforementioned advantages. However, as 3D printing technology continues to evolve, we expect that the accessibility and material flexibility of PBF, MJ, and SLA methods will improve, accelerating the progress and broadening the horizon of the 3D printed energy devices.

Third, there is a strong demand for multimaterial 3D printing, as the challenge of producing the entire energy device in a single printing step still remains. Generally, energy devices comprise more than three individual constituent materials, such as functional materials (piezoelectric material, thermoelectric material, etc.), conductive electrodes, and dielectric packaging materials. However, most existing 3D printing methods are based on the single material printing process, making it difficult to manufacture the complete energy device in a single step. Multi-material 3D printing enables the printing of different types of constituent materials in sequence^17,131^, facilitating the direct integration of the devices and expanding the capabilities beyond what conventional single-material 3D printing can offer. Furthermore, the incorporation of two different printing processes, referred to as hybrid printing technology, holds promise as an alternative method to fabricate the energy devices in a single step. In a hybrid printing system, two complementary printing methods can seamlessly produce multiple components, either continuously or alternately, within a single process^132^, facilitating the production of integrated energy devices.

Fourth, 3D printing processes tend to introduce micro/nano pores in the printed structures due to the layer-by-layer nature of the processes. Although porous structures offer benefits such as increased specific surface area, which enhances power and energy density in certain electrochemical energy devices, they typically exhibit poor mechanical properties. Moreover, the layer-by-layer process often results in weakly bonded interfaces between adjacent layers, further contributing to suboptimal mechanical performances. Therefore, additional research should be conducted to enhance the mechanical properties of 3D-printed energy devices, particularly to meet the strict robustness requirements of industrial applications.

Furthermore, for practical applications, it is also essential to address multiple factors, including improving printing resolution, reducing printing time, achieving better scalability for the production of larger device structures, and gaining a fundamental understanding of the physical and chemical behaviors of printing materials during the manufacturing process. Despite these challenges, 3D printing technologies have the strong potential to overcome the barriers impending the advancement of energy devices. We envision that with the continuous advancement of 3D printing processes, materials, and designs, 3D-printed energy devices with enhanced mechanical property, integrability, high resolution, and exceptional electrical performance will eventually find widespread use across various fields.
